# Disinfection By-Product Exposures and the Risk of Specific Cardiac Birth Defects

**DOI:** 10.1289/EHP103

**Published:** 2016-08-12

**Authors:** J. Michael Wright, Amanda Evans, John A. Kaufman, Zorimar Rivera-Núñez, Michael G. Narotsky

**Affiliations:** 1 National Center for Environmental Assessment (NCEA), Office of Research and Development (ORD), U.S. Environmental Protection Agency (EPA), Cincinnati, Ohio, USA; 2 School of Osteopathic Medicine, Campbell University, Lillington, North Carolina, USA; 3 ASPPH/EPA Environmental Health Fellowship Program, hosted by NCEA, ORD, U.S. EPA, Cincinnati, Ohio, USA; 4 Radiation Oncology, Rutgers Cancer Institute of New Jersey, New Brunswick, New Jersey, USA; 5 National Health and Environmental Effects Research Laboratory, ORD, U.S. EPA, Research Triangle Park, North Carolina, USA

## Abstract

**Background:**

Epidemiological studies suggest that women exposed to disinfection by-products (DBPs) have an increased risk of delivering babies with cardiovascular defects (CVDs).

**Objective:**

We examined nine CVDs in relation to categorical DBP exposures including bromoform, chloroform, dibromochloromethane (DBCM), bromodichloromethane (BDCM), monobromoacetic acid (MBAA), dichloroacetic acid (DCAA), trichloroacetic acid (TCAA), and summary DBP measures (HAA5, THMBr, THM4, and DBP9).

**Methods:**

We calculated adjusted odds ratios (aORs) in a case–control study of birth defects in Massachusetts with complete quarterly 1999–2004 trihalomethane (THM) and haloacetic acid (HAA) data. We randomly matched 10 controls each to 904 CVD cases based on week of conception. Weight-averaged aggregate first-trimester DBP exposures were assigned to individuals based on residence at birth.

**Results:**

We detected associations for tetralogy of Fallot and the upper exposure categories for TCAA, DCAA, and HAA5 (aOR range, 3.34–6.51) including positive exposure–response relationships for DCAA and HAA5. aORs consistent in magnitude were detected between atrial septal defects and bromoform (aOR = 1.56; 95% CI: 1.01, 2.43), as well as DBCM, chloroform, and THM4 (aOR range, 1.26–1.67). Ventricular septal defects (VSDs) were associated with the highest bromoform (aOR = 1.85; 95% CI: 1.20, 2.83), MBAA (aOR = 1.81; 95% CI: 0.85, 3.84), and DBCM (aOR = 1.54; 95% CI: 1.00, 2.37) exposure categories.

**Conclusions:**

To our knowledge, this is the first birth defect study to develop multi-DBP adjusted regression models as well as the first CVD study to evaluate HAA exposures and the second to evaluate bromoform exposures. Our findings, therefore, inform exposure specificity for the consistent associations previously reported between THM4 and CVDs including VSDs.

**Citation:**

Wright JM, Evans A, Kaufman JA, Rivera-Núñez Z, Narotsky MG. 2017. Disinfection by-product exposures and the risk of specific cardiac birth defects. Environ Health Perspect 125:269–277; http://dx.doi.org/10.1289/EHP103

## Introduction

Cardiovascular defects (CVDs) are the most common type of birth defect, with an estimated incidence in the United States of 8 in 1,000 births (Go et al. 2013). CVDs can lead to a higher infant mortality rate among newborns, and their etiology is often unknown (Kurinczuk et al. 2010; Lee et al. 2001). Some environmental hazards are known teratogens, such as methylmercury and radiation, but the existing evidence for associations between drinking-water contaminants and birth defects is mixed (Brent and Beckman 1990; Nieuwenhuijsen et al. 2009). Although the risk of CVDs in relation to specific disinfection by-products (DBPs) remains unclear, there is some evidence for associations between CVDs and a summary measure of trihalomethanes (THMs) called THM4 [i.e., sum of chloroform, bromoform, bromodichloromethane (BDCM), and dibromochloromethane (DBCM)]. Nieuwenhuijsen et al. (2009) conducted a meta-analysis of 5 case–control studies and 10 retrospective cohort studies using THM concentration data or less direct exposure measures (e.g., treatment type/source water). The authors reported a small but not statistically significant odds ratio of 1.16 [95% confidence interval (CI): 0.98, 1.37] for major CVDs among those with high DBP exposures. A prospective cohort study from Lithuania based on first-trimester internal DBP dose estimates showed elevated odds ratios for CVDs, with exposure–response relationships detected for THM4, BDCM, and chloroform tertiles (Grazuleviciene et al. 2013). Slightly elevated odds ratios were noted in a study from England of major CVDs and the sum of three brominated THMs (i.e., THMBr) ≥ 20 versus < 10 μg/L [adjusted odds ratio (aOR) = 1.13; 95% CI: 0.93, 1.37] and between bromoform ≥ 4 versus < 2 μg/L (aOR = 1.18; 95% CI: 1.00, 1.39) and a restricted group of major CVDs (Nieuwenhuijsen et al. 2008). Stronger associations (aOR = 1.62; 95% CI: 1.04, 2.51) have also been reported for CVDs and THM4 ≥ 130 versus < 60 μg/L in an Australian population using heavily brominated water (Chisholm et al. 2008). Studies with rats exposed to brominated THMs were negative for CVDs (Christian et al. 2001; Ruddick et al. 1983); however, one study examining monobromoacetic acid (MBAA) exposure reported increased incidence of CVDs (Randall et al. 1991). Another study in rats reported CVDs [e.g., ventricular septal defect (VSD), levocardia, right-sided aortic arch, and ductus arteriosus] following exposure to bromochloroacetonitrile (Christ et al. 1995).

The strongest and most consistent associations reported in epidemiological studies of birth defects and DBPs have been for VSDs. Similar to an earlier meta-analysis by Hwang et al. (2008), Nieuwenhuijsen et al. (2009) found a consistent excess risk for VSDs (OR = 1.59; 95% CI: 1.21, 2.07) in three studies using various THM and chlorinated water exposure measures (OR range: 1.43–1.81). An earlier study, not included in the published meta-analyses, did not report associations between THM exposure and VSD (Bove et al. 1995). A more recent Italian study also did not show an increased risk of VSD for chlorine dioxide DBPs, including chlorite and chlorate (Righi et al. 2012).

Exposure assessment limitations in epidemiological studies remain a critical challenge in evaluating causality in reported associations between DBPs and various reproductive outcomes given limited spatial and temporal resolution of monitoring data and the lack of direct exposure measures. Previous epidemiological studies of CVDs have not examined exposures beyond THMs and chlorine dioxide DBPs. This remains a key limitation given that animal developmental toxicity studies of THMs are generally negative for teratogenicity (Graves et al. 2001) and that the THMs may be poor surrogates for complex DBP mixtures in chlorinated drinking-water systems. Furthermore, although haloacetic acids (HAAs) have not been examined in epidemiological studies of CVDs and DBPs to date, toxicological studies in rats have shown increased incidence of VSDs and conotruncal defects following exposure to dichloroacetic acid (DCAA) and trichloroacetic acid (TCAA) (Epstein et al. 1992; Johnson et al. 1998; Smith et al. 1989, 1992). Most epidemiological studies of DBPs also have limited exposure contrasts and insufficient statistical power to detect rare outcomes like CVDs. This may preclude the ability to detect statistically significant associations that are small in magnitude and to examine exposure–response relationships for individual CVDs and specific DBP species. To address some of these exposure assessment limitations and expand the scope of the birth defect and DBP combinations that have been previously examined, we assessed the risk of CVDs in relation to weighted first-trimester exposure estimates for nine individual DBPs and four DBP mixture surrogates.

## Methods

### Study Population

We conducted a case–control study of CVD cases in 68 Massachusetts towns with populations > 500 with complete THM4, HAA5 [i.e., sum of monochloroacetic acid (MCAA), DCAA, TCAA, MBAA, and dibromoacetic acid (DBAA)], water source and disinfection data from 1999 to 2004. We restricted the analysis to nonchromosomal congenital anomalies of the heart and circulatory system (*n* = 904 cases) and individually matched 10 controls per each case randomly selected (without replacement) from all live births in Massachusetts based on week of conception, for a total study population of 9,944.

### Outcome Data

Birth records from 2000 through 2004 were provided by the Massachusetts Department of Public Health and the Massachusetts Birth Defects Monitoring Program. The Massachusetts birth defect registry system collects data from 53 birthing hospitals, 1 tertiary care and 1 specialty hospital in Massachusetts, and 1 Rhode Island birth hospital and 1 Rhode Island tertiary care hospital near the border of these two states. The registry system uses various data sources to ascertain and verify cases including birth certificates, fetal and infant death certificates, hospital discharge reports, hospital nurseries and neonatal units, and hospital surgical and pathology departments. This research was based on birth records data that did not contain personal identifiable information; therefore, institutional review board approval was not obtained nor was informed consent necessary because potential risk was considered to be minimal and no direct contact with study subjects occurred.

Birth defect cases were diagnosed up to age 1 year. Both cases and controls were singleton live births who weighed at least 350 g and were between 22 and 44 gestational weeks. Cases were identified based on the *International Classification of Diseases, 9th Revision* (ICD-9). These included atrial septal defect (ASD; ICD-9 code 745.5), VSD (745.4), pulmonary stenosis (746.02), tetralogy of Fallot (TOF; 745.2), and transposition of the great arteries (TGA; 745.10, 745.11, 745.12, 745.19). We also examined birth defect group combinations including all congenital anomalies of the heart and circulatory system (All CVD; 745–747), and conotruncal heart defects (745.0, 745.10, 745.11, 745.12, 745.2). Gestational age was derived from clinical estimates according to birth records and was subtracted from date of birth to determine week of conception for matching purposes.

### Exposure Assessment

We linked town-level drinking-water source, disinfection treatment, and DBP data based on quarterly sampling (1999–2004) to birth records by town of residence and month of birth. The exposure data were supplied by the Massachusetts Department of Environmental Protection and individual public water utilities. Exposures were estimated for specific DBPs as well as summary measures of DBP mixtures including THM4, HAA5, DBP9 (i.e., sum of THM4 and HAA5), and THMBr. We categorized maternal DBP exposure levels for the summary and individual DBP measures into tertiles, quartiles, or quintiles based on the distribution of the available data. Due to a paucity of occurrence data, MCAA was dichotomized at the 97.5th percentile (0.04 μg/L), and bromoform (0.26 μg/L), DBCM (0.47 μg/L), and DBAA (1.53 μg/L) were dichotomized at the upper decile. Births in the lowest DBP exposure category served as the referent for comparison with the upper categories. This categorical approach allowed for evaluation of nonlinear relationships and potential effect measure modification using stratified analyses.

We averaged first-trimester DBP exposures across all sample locations within a public drinking-water system based on quarterly monitoring data assigned to maternal ZIP codes for place of residence at birth. The first-trimester DBP exposure scores were derived from the month of birth of the study participants and the timing of quarterly DBP samples with weighted averages calculated proportionally for multiple quarters that overlapped the first trimester. For example, an infant of 38 gestational weeks born in January of 2000 would have 2 first-trimester weeks that occurred in quarter 1 of 1999 and the remaining 11 weeks occurring in quarter 2 of 1999. Thus, their corresponding exposure score would be as follows: (0.15 times the DBP concentration for quarter 1 of 1999) + (0.85 times the DBP concentration for quarter 2 of 1999). In addition, residents relying on untreated groundwater (e.g., private wells) were assigned DBP concentrations of zero.

### Statistical Analysis

SAS (version 9.4; SAS Institute, Inc., Cary, NC) was used for the statistical analysis. We used Spearman correlation coefficients to compare the summary and individual DBP measures. Statistical significance was based on α ≤ 0.05. We used conditional logistic regression to estimate aORs and 95% CIs for each of the DBP exposure categories. Given the extensive amount of available covariate data, we used a change-in-estimate approach (> 10% change) to identify confounding variables. These covariates included type of water source and treatment, infant sex, infant birth weight, maternal weight gain, maternal race, maternal age, maternal education, marital status (not married vs. married, including within 300 days before birth), maternal smoking (cigarettes/day during pregnancy), parity, number of previous terminations, prenatal care source payment type, income, and various clinical factors (e.g., abruptio placenta, anemia, cardiac disease, chronic or gestational diabetes, chronic or gestational hypertension, eclampsia, hemoglobinopathy, hepatitis, hydramnios/oligohydramnios, incompetent cervix, labor/delivery complication, labor induction, lung disease, lupus, other maternal reproductive risk factors, pharmaceutical inhibition of labor, previous infant > 4,000 g, previous infant with birth defect, previous premature or small-for-gestational-age infant, premature or prolonged rupture of membrane, renal disease, Rh sensitization, rubella infection, seizure disorder, sickle cell anemia, uterine bleeding). We examined a categorical health index (values ranging from 0 to 5) that included presence of hydramnios/oligohydramnios, chronic hypertension, gestational hypertension, gestational diabetes, and nongestational diabetes. We also evaluated adequacy of prenatal care by the Kotelchuck Index (Kotelchuck 1994), which integrates information on the timing (i.e., first trimester vs. later during pregnancy) of initiation of prenatal care and the number of prenatal visits (< 9, 9–11, 12, 13–15, > 15) from when prenatal care began until delivery. These two individual prenatal covariate constituents were examined separately as confounders, as well as part of the Kotelchuck Index.

All of the covariates were based on the individual-level data obtained from birth records except for income and DBP data, as well as the information on type of water source and type of water treatment. Median household income for maternal residence at birth were obtained from the 2000 U.S. Census (Geolytics, Inc., East Brunswick, NJ). Aggregate-level income covariates were examined at three spatial scales: town, ZIP code, and census tract. We created a socioeconomic status index based on ZIP code–level income data combined with mother’s highest education level and prenatal care source of payment. The results presented here are also based on multi-pollutant models by adjusting for THM4 in all of the HAA models and adjusting for HAA5 in the THM models. Effect measure modification by infant sex was examined by stratification for the outcome and DBPs with most consistent and largest aORs (i.e., TOF and VSDs). We conducted a sensitivity analysis of the impact of multiple birth defects, as well as an analysis of the All CVD category excluding patent ductus arteriosus.

## Results

Among all reported births from 2000 to 2004 in Massachusetts, there were 904 (45% of the total birth defects) CVDs. The most common CVDs were ASDs (41%) and VSDs (37%). Forty-three percent (*n* = 390) of the birth defects examined here were isolated CVDs, whereas 57% (*n* = 514) of the cases had multiple defects. Among the 514 cases with multiple defects, 377 (73%) of them were CVDs only. As shown in Table 1, cases and controls were similar across most study characteristics, with minor exceptions noted for health index scores and the Kotelchuck Index for adequacy of prenatal care. Controls were more likely to be born to healthier mothers based on our health index score.

**Table 1:** Study characteristics of cardiovascular defect (CVD) cases and controls [*n* (%)].

Characteristic	Study population	Cases	Controls
Total births	9,944 (100)	904 (100)	9,040 (100)
Infant sex
Male	5,119 (51.5)	468 (51.8)	4,651 (51.4)
Female	4,825 (48.5)	436 (48.2)	4,389 (48.6)
Maternal age (years)
≤ 20	907 (9.1)	82 (9.1)	825 (9.1)
> 20–25	1,733 (17.4)	169 (18.7)	1,564 (17.3)
> 25–30	2,648 (26.6)	223 (24.7)	2,425 (26.8)
> 30–35	3,008 (30.3)	273 (30.2)	2,735 (30.3)
> 35–40	1,384 (13.9)	122 (13.5)	1,262 (14.0)
> 40	264 (2.7)	35 (3.9)	229 (2.5)
Maternal race
White	6,614 (66.6)	589 (65.2)	6,025 (66.6)
African American	995 (10.0)	114 (12.6)	881 (9.7)
Asian	747 (7.5)	53 (5.9)	694 (7.7)
American Indian	22 (0.2)	5 (0.6)	17 (0.2)
Other	1,561 (15.7)	143 (15.8)	1,418 (15.7)
Maternal education
Below high school graduate/GED	1,166 (11.7)	119 (13.2)	1,047 (11.6)
High school graduate/GED	2,689 (27.0)	244 (27.0)	2,445 (27.0)
Some college or associates/technical degree	2,076 (20.9)	184 (20.4)	1,892 (20.9)
College or higher	4,013 (40.4)	357 (39.5)	3,656 (40.4)
Marital status
Married	6,886 (69.3)	602 (66.6)	6,284 (69.5)
Unmarried	3,041 (30.6)	300 (33.2)	2,741 (30.3)
Missing	17 (0.2)	2 (0.2)	15 (0.2)
Number of previous births
0	4,508 (45.4)	413 (45.7)	4,095 (45.3)
1	3,269 (32.9)	287 (31.7)	2,982 (33.0)
≥ 2	2,154 (21.7)	204 (22.6)	1,950 (21.6)
Maternal weight gain during pregnancy (lb)
< 0	112 (1.1)	14 (1.5)	98 (1.1)
0–25	3,867 (39.1)	365 (40.4)	3,502 (38.7)
25–50	5,557 (56.2)	489 (54.1)	5,068 (56.1)
> 50	354 (3.6)	30 (3.3)	324 (3.6)
Maternal smoking during pregnancy (no. of cigarettes/day during pregnancy)	
0	9,165 (92.2)	830 (91.8)	8,335 (92.2)
1–5	327 (3.3)	22 (2.4)	305 (3.4)
6–10	289 (2.9)	34 (3.8)	255 (2.8)
> 10	163 (1.6)	18 (2.0)	145 (1.6)
Prenatal care adequacy (Kotelchuck Index)
No prenatal care	48 (0.5)	6 (0.7)	42 (0.5)
Inadequate	886 (8.9)	92 (10.2)	794 (8.8)
Intermediate	713 (7.2)	41 (4.5)	672 (7.4)
Adequate	4,583 (46.1)	386 (42.7)	4,197 (46.4)
Adequate plus	3,714 (37.4)	379 (41.9)	3,335 (36.9)
Prenatal care source of payment
Public	2,611 (26.3)	277 (30.6)	2,334 (25.8)
Private	6,556 (65.9)	566 (62.6)	5,990 (66.3)
Other	777 (7.8)	61 (6.7)	716 (7.9)
Median household income (based on ZIP code from 2000)
$12,307–36,836	2,492 (25.1)	249 (27.5)	2,243 (24.8)
> $36,836–45,654	2,303 (23.2)	215 (23.8)	2,088 (23.1)
> $45,654–57,815	2,524 (25.4)	219 (24.2)	2,305 (25.5)
> $57,815–153,918	2,625 (26.4)	221 (24.4)	2,404 (26.6)
Note: GED, General Educational Development.			

As shown in Table 2, median and interquartile ranges (μg/L) for the nine predominant DBP metrics were as follows: DBP9 (69.6; 44.1–92.1), THM4 (44.5; 29.3–61.4), chloroform (36.1; 17.4–51.0), THMBr (6.8; 4.8–10.2), BDCM (6.1; 4.5–8.4), DBCM (0.6; 0–1.6), HAA5 (22.4; 11.3–31.2), TCAA (11.0; 5.4–16.3), and DCAA (10.4; 5.3–13.7). We observed Spearman correlation coefficients ≥ 0.9 for the following: DBP9 with THM4, HAA5, and chloroform; HAA5 with TCAA and DCAA; THM4 with chloroform; and THMBr with BDCM (see Table S1). We observed correlations between 0.7 and 0.9 for the following: DBP9 with TCAA and DCAA; HAA5 with THM4 and chloroform; THM4 with TCAA and CCAA; DBCM with BDCM and THMBr; chloroform with TCAA and DCAA; and TCAA with DCAA. The strongest correlations among the individual brominated species were found between DBCM and BDCM (*r* = 0.6) and DBAA (*r* = 0.4), as well as between bromoform and DBCM (*r* = 0.5) and DBAA (*r* = 0.3).

**Table 2:** First-trimester disinfection by-product (DBP) (μg/L) exposure levels for the study population.

DBP metric	Mean ± SD	25th %	50th %	75th %	90th %	95th %	Maximum
DBP9	65.07 ± 36.49	44.14	69.57	92.14	107.8	117.03	181.59
THM4	42.67 ± 24.05	29.26	44.53	61.37	72.71	76.63	125.32
THMBr	8.28 ± 6.51	4.75	6.75	10.17	17.87	21.20	42.48
Chloroform	34.39 ± 21.56	17.38	36.07	51.03	62.65	67.15	98.99
BDCM	6.85 ± 5.05	4.47	6.12	8.36	13.04	16.35	37.38
DBCM	1.32 ± 2.01	0.00	0.56	1.57	3.97	5.98	14.53
Bromoform	0.12 ± 0.46	0.00	0.00	0.00	0.26	0.86	7.06
HAA5	22.40 ± 14.89	11.34	22.38	31.18	42.52	47.49	100.00
TCAA	11.53 ± 8.42	5.40	11.01	16.30	22.16	26.37	73.39
DCAA	9.90 ± 6.61	5.30	10.38	13.67	18.61	21.29	38.89
MCAA	0.83 ± 3.05	0.00	0.05	0.84	1.53	2.13	62.39
DBAA	0.17 ± 0.70	0.00	0.00	0.00	0.47	1.06	21.78
MBAA	0.02 ± 0.26	0.00	0.00	0.00	0.00	0.00	10.63
Note: %, percentile; DBP9, sum of chloroform, bromodichloromethane (BDCM), dibromochloromethane (DBCM), bromoform, monochloroacetic acid (MCAA), dichloroacetic acid (DCAA), trichloroacetic acid (TCAA), monobromoacetic acid (MBAA), and dibromoacetic acid (DBAA); HAA5, sum of MCAA, DCAA, TCAA, MBAA, and DBAA; THM4, sum of chloroform, BDCM, DBCM, and bromoform; THMBr*,* sum of BDCM, DBCM, and bromoform.							

We did not detect an increased risk for the overall CVD group (i.e., All CVD) and first-trimester THM4 exposures, but there was a statistically significant association for dichotomized bromoform exposures (aOR = 1.43; 95% CI: 1.10, 1.86) and increased risks in the upper two DCAA quartiles (aOR range, 1.21–1.23) and upper three HAA5 quintiles (aOR range, 1.18–1.42) (Table 3). We saw consistent evidence of associations for conotruncal defects in the upper three HAA quintiles (aOR range, 1.77–3.76) and the upper three TCAA quartiles (aOR range, 1.95–2.13). We detected stronger associations for TOF and the upper exposure categories for TCAA, DCAA, and HAA5 (aOR range, 3.34–6.51) including positive exposure–response relationships for DCAA and HAA5. We detected statistically significant associations for TGA and bromoform exposure (aOR = 2.42; 95% CI: 1.12, 5.23) and for the two intermediate HAA5 quintiles (aOR range, 4.26–4.54); the upper quintile was limited by a small number of cases (*n* = 9). Inverse associations among the upper exposure categories were detected between TGA and DBP9 and between TOF and chloroform, BDCM, THMBr, and THM4.

**Table 3:** Adjusted odds ratios (aORs) between disinfection by-product (DBP) exposures and congenital anomalies of the heart and circulatory system (All CVD), conotruncal heart defects, transposition of the great arteries (TGA), and tetralogy of Fallot (TOF).

DBP metrics (μg/L)^*a*^	All CVD^*c*^		Conotruncal^*d*^		TGA^*e*^		TOF^*f*^	
	Cases^*b*^ (*n*)	aOR (95% CI)	Cases^*b*^ (*n*)	aOR (95% CI)	Cases^*b*^ (*n*)	aOR (95% CI)	Cases^*b*^ (*n*)	aOR (95% CI)
THM4^*g*^
> 23.05–38.05	184	1.04 (0.72, 1.51)	37	0.96 (0.43, 2.14)	21	1.70 (0.58, 4.95)	17	0.32 (0.10, 1.05)
> 38.05–50.41	188	1.02 (0.67, 1.54)	45	1.19 (0.50, 2.82)	26	1.87 (0.57, 6.08)	19	0.51 (0.15, 1.74)
> 50.41–65.27	192	1.04 (0.67, 1.62)	32	0.68 (0.27, 1.74)	13	1.02 (0.28, 3.82)	19	0.31 (0.08, 1.21)
> 65.27–125.32	167	0.81 (0.51, 1.31)	31	0.50 (0.17, 1.48)	15	0.81 (0.17, 3.84)	16	0.19 (0.04, 0.88)
THMBr^*g*^
> 4.17–6.04	173	0.99 (0.65, 1.51)	39	0.72 (0.32, 1.60)	14	0.45 (0.13, 1.61)	25	1.00 (0.34, 2.98)
> 6.04–7.80	195	1.05 (0.71, 1.55)	39	0.60 (0.27, 1.30)	26	0.98 (0.35, 2.74)	12	0.23 (0.06, 0.91)
> 7.80–11.51	176	0.97 (0.66, 1.42)	32	0.47 (0.21, 1.02)	14	0.49 (0.16, 1.49)	18	0.45 (0.14, 1.39)
> 11.51–42.48	188	1.02 (0.71, 1.46)	34	0.39 (0.19, 0.80)	19	0.55 (0.20, 1.51)	16	0.26 (0.09, 0.76)
Chloroform^*g*^
> 12.07–29.99	181	1.05 (0.73, 1.50)	33	1.22 (0.56, 2.65)	19	1.49 (0.53, 4.24)	14	0.50 (0.16, 1.53)
> 29.99–42.17	197	1.03 (0.68, 1.55)	46	1.46 (0.62, 3.47)	25	1.69 (0.52, 5.50)	21	0.76 (0.22, 2.69)
> 42.17–55.41	184	0.94 (0.60, 1.47)	37	1.42 (0.53, 3.79)	15	1.28 (0.33, 4.95)	22	0.81 (0.20, 3.29)
> 55.41–98.99	167	0.77 (0.47, 1.25)	29	0.63 (0.21, 1.93)	15	0.55 (0.11, 2.83)	14	0.33 (0.07, 1.64)
Bromodichloromethane (BDCM)^*g*^
> 4.95–7.55	325	0.97 (0.73, 1.28)	61	0.73 (0.40, 1.34)	36	0.83 (0.37, 1.86)	24	0.49 (0.19, 1.24)
> 7.55–37.38	286	0.93 (0.72, 1.19)	51	0.49 (0.29, 0.82)	24	0.57 (0.27, 1.22)	28	0.38 (0.17, 0.82)
Dibromochloromethane (DBCM)^*g*^
> 3.93–14.53	87	1.01 (0.77, 1.33)	16	0.68 (0.36, 1.29)	10	1.01 (0.43, 2.39)	7	0.54 (0.20, 1.41)
Bromoform^*g*^
> 0.26–7.06	109	1.43 (1.10, 1.86)	25	1.30 (0.72, 2.33)	16	2.42 (1.12, 5.23)	10	1.14 (0.45, 2.87)
HAA5^*h*^
> 8.17–19.33	160	0.99 (0.69, 1.42)	38	1.18 (0.53, 2.66)	17	0.86 (0.28, 2.64)	21	2.13 (0.53, 8.65)
> 19.33–25.79	219	1.42 (0.95, 2.12)	49	3.19 (1.29, 7.89)	29	4.54 (1.26, 16.41)	21	4.98 (1.02, 24.35)
> 25.79–33.97	172	1.35 (0.87, 2.10)	37	3.76 (1.38, 10.24)	19	4.26 (1.02, 17.87)	17	5.88 (1.06, 32.57)
> 33.97–100.00	174	1.18 (0.75, 1.86)	24	1.77 (0.63, 4.97)	9	1.10 (0.23, 5.37)	16	6.51 (1.23, 34.59)
Trichloroacetic acid (TCAA)^*h*^
> 5.23–11.09	234	1.17 (0.86, 1.60)	59	2.09 (1.02, 4.29)	30	1.38 (0.53, 3.56)	28	2.72 (0.91, 8.13)
> 11.09–16.38	237	1.11 (0.76, 1.62)	44	2.13 (0.89, 5.14)	22	1.39 (0.40, 4.80)	22	4.30 (1.09, 16.88)
> 16.38–73.39	208	1.03 (0.69, 1.55)	36	1.95 (0.77, 4.92)	18	1.07 (0.29, 3.86)	19	3.89 (0.97, 15.66)
Dichloroacetic acid (DCAA)^*h*^
> 5.18–10.44	219	1.04 (0.78, 1.40)	48	1.07 (0.57, 2.04)	27	0.96 (0.41, 2.23)	22	1.39 (0.50, 3.88)
> 10.44–13.85	241	1.21 (0.86, 1.70)	52	1.60 (0.76, 3.36)	28	1.21 (0.43, 3.41)	24	3.08 (0.92, 10.34)
> 13.85–38.89	220	1.23 (0.85, 1.78)	34	1.16 (0.50, 2.70)	12	0.50 (0.14, 1.77)	21	3.34 (0.90, 12.43)
Monochloroacetic acid (MCAA)^*h*^
> 1.53–62.39	84	1.06 (0.80, 1.40)	9	0.85 (0.41, 1.77)	6	1.10 (0.43, 2.77)	4	0.83 (0.27, 2.54)
Monobromoacetic acid (MBAA)^*h*^
> 0.04–10.63	24	1.17 (0.66, 2.07)	4	0.77 (0.17, 3.48)	1	0.47 (0.06, 3.97)	3	0.80 (0.08, 7.48)
Dibromoacetic acid (DBAA)^*h*^
> 0.47–21.78	73	0.81 (0.61, 1.08)	19	0.72 (0.39, 1.33)	11	0.96 (0.41, 2.26)	7	0.59 (0.23, 1.54)
DBP9
> 33.07–59.95	187	1.37 (0.95, 1.97)	37	1.11 (0.52, 2.37)	21	1.22 (0.45, 3.31)	17	0.80 (0.24, 2.63)
> 59.95–79.13	196	1.30 (0.87, 1.92)	49	1.12 (0.51, 2.49)	24	0.87 (0.29, 2.61)	24	1.15 (0.32, 4.08)
> 79.13–97.67	194	1.37 (0.92, 2.05)	36	1.23 (0.54, 2.78)	19	1.02 (0.34, 3.11)	18	1.39 (0.39, 5.01)
> 97.67–181.59	160	0.96 (0.63, 1.46)	24	0.36 (0.14, 0.95)	10	0.08 (0.01, 0.46)	14	0.94 (0.23, 3.79)
Note: CI, confidence interval; DBP9, sum of chloroform, BDCM, DBCM, bromoform, MCAA, DCAA, TCAA, MBAA, and DBAA; HAA5, sum of MCAA, DCAA, TCAA, MBAA, and DBAA; THM4, sum of chloroform, BDCM, DBCM, and bromoform; THMBr, sum of BDCM, DBCM, and bromoform. ^***a***^For each DBP metric, the referent for each model includes exposure scores of zero up to the lower bound of the lowest exposure category.^***b***^The numbers represent the case distribution across exposure groups before modeling.^***c***^Models adjusted for the type of water source and treatment, infant birth weight, town-level income quartile, number of prenatal care visits (< 9, 9–11, 12, 13–15, > 15), health index (gestational diabetes, non-gestational diabetes, chronic hypertension, gestational hypertension, and hydramnios/oligohydramnios), and other maternal reproductive risk factors.^***d***^Models adjusted for the type of water source and treatment, health index (gestational diabetes, non-gestational diabetes, chronic hypertension, gestational hypertension, and hydramnios/oligohydramnios), and other maternal reproductive risk factors.^***e***^Models adjusted for the type of water source and treatment, number of prenatal care visits (< 9, 9–11, 12, 13–15, > 15), ZIP code–level income quartile, trimester prenatal care began (first, after first), health index (gestational diabetes, nongestational diabetes, chronic hypertension, gestational hypertension, and hydramnios/oligohydramnios), and other maternal reproductive risk factors.^***f***^Models adjusted for the type of water source and treatment, infant birth weight, ZIP code–level income quartile, trimester prenatal care began (first, after first), health index (gestational diabetes, nongestational diabetes, chronic hypertension, gestational hypertension, and hydramnios/oligohydramnios), and other maternal reproductive risk factors.^***g***^Models also include adjustment for HAA5 concentrations.^***h***^Models also include adjustment for THM4 concentrations.								

Increased aORs were detected for pulmonary stenosis and bromoform exposures (aOR = 2.66; 95% CI: 1.30, 5.43), the upper two DCAA quartiles (aOR range, 1.65–2.02), as well as a positive exposure–response relationship for TCAA (aOR range, 1.47–3.45) (Table 4). aORs consistent in magnitude were detected for ASDs and bromoform (aOR = 1.56; 95% CI: 1.01, 2.43), DBCM (aOR = 1.26; 95% CI: 0.81, 1.97), and for each THM4 (aOR range, 1.28–1.59) and chloroform quintile (aOR range, 1.38–1.67).

**Table 4:** Adjusted odds ratios (aORs) between disinfection by-product (DBP) exposures and atrial septal defects (ASDs), ventricular septal defects (VSDs), and pulmonary stenosis (PS).

DBP metrics (μg/L)^*a*^	ASD^*c*^		VSD^*d*^		PS^*e*^	
	Cases^*b*^ (*n*)	aOR (95% CI)	Cases^*b*^ (*n*)	aOR (95% CI)	Cases^*b*^ (*n*)	aOR (95% CI)
THM4^*f*^
> 23.05–38.05	78	1.31 (0.70, 2.46)	61	1.39 (0.72, 2.69)	24	0.61 (0.23, 1.61)
> 38.05–50.41	72	1.34 (0.67, 2.66)	80	1.77 (0.87, 3.59)	26	0.70 (0.24, 2.04)
> 50.41–65.27	86	1.59 (0.77, 3.26)	73	1.70 (0.80, 3.63)	18	0.35 (0.11, 1.19)
> 65.27–125.32	67	1.28 (0.58, 2.84)	58	1.57 (0.70, 3.53)	24	0.49 (0.14, 1.75)
THMBr^*f*^
> 4.17–6.04	78	0.73 (0.35, 1.52)	56	0.81 (0.39, 1.68)	21	0.84 (0.27, 2.63)
> 6.04–7.80	83	1.10 (0.58, 2.09)	73	0.79 (0.41, 1.55)	25	0.65 (0.24, 1.77)
> 7.80–11.51	67	0.99 (0.53, 1.88)	55	0.89 (0.47, 1.69)	24	0.46 (0.16, 1.29)
> 11.51–42.48	76	1.14 (0.63, 2.06)	82	1.34 (0.73, 2.46)	25	0.91 (0.36, 2.29)
Chloroform^*f*^
> 12.07–29.99	75	1.38 (0.73, 2.60)	61	1.05 (0.58, 1.90)	27	0.74 (0.29, 1.89)
> 29.99–42.17	86	1.67 (0.83, 3.38)	74	0.86 (0.43, 1.72)	28	0.73 (0.24, 2.27)
> 42.17–55.41	76	1.50 (0.69, 3.26)	75	1.00 (0.47, 2.12)	19	0.40 (0.12, 1.40)
> 55.41–98.99	68	1.42 (0.61, 3.27)	57	0.79 (0.35, 1.80)	20	0.37 (0.10, 1.44)
Bromodichloromethane (BDCM)^*f*^	
> 4.95–7.55	139	1.19 (0.75, 1.90)	112	0.81 (0.50, 1.31)	39	0.59 (0.29, 1.23)
> 7.55–37.38	111	1.13 (0.74, 1.72)	116	1.21 (0.79, 1.85)	37	0.69 (0.35, 1.37)
Dibromochloromethane (DBCM)^*f*^	
> 3.93–14.53	36	1.26 (0.81, 1.97)	40	1.54 (1.00, 2.37)	10	0.96 (0.41, 2.25)
Bromoform^*f*^
> 0.26–7.06	46	1.56 (1.01, 2.43)	43	1.85 (1.20, 2.83)	20	2.66 (1.30, 5.43)
HAA5^*g*^
> 8.17–19.33	61	0.50 (0.28, 0.90)	50	0.66 (0.37, 1.20)	20	0.72 (0.26, 1.98)
> 19.33–25.79	103	0.91 (0.48, 1.74)	74	1.00 (0.51, 1.94)	29	1.34 (0.42, 4.26)
> 25.79–33.97	65	0.63 (0.31, 1.29)	63	1.01 (0.49, 2.08)	24	1.48 (0.41, 5.36)
> 33.97–100.00	65	0.41 (0.19, 0.86)	78	1.02 (0.49, 2.12)	21	1.06 (0.28, 4.06)
Trichloroacetic acid (TCAA)^*g*^
> 5.23–11.09	100	0.69 (0.42, 1.15)	81	0.90 (0.55, 1.50)	29	1.47 (0.57, 3.74)
> 11.09–16.38	97	0.58 (0.31, 1.09)	83	0.81 (0.44, 1.51)	32	2.48 (0.82, 7.46)
> 16.38–73.39	79	0.36 (0.18, 0.70)	86	0.79 (0.41, 1.50)	29	3.45 (1.04, 11.39)
Dichloroacetic acid (DCAA)^*g*^
> 5.18–10.44	85	0.78 (0.48, 1.28)	72	0.84 (0.51, 1.39)	23	0.93 (0.38, 2.26)
> 10.44–13.85	103	0.88 (0.50, 1.54)	92	1.28 (0.72, 2.25)	40	2.02 (0.74, 5.50)
> 13.85–38.89	89	0.75 (0.41, 1.37)	88	1.18 (0.65, 2.14)	27	1.65 (0.52, 5.22)
Monochloroacetic acid (MCAA)^*g*^	
> 1.53–62.39	31	1.08 (0.67, 1.73)	35	1.27 (0.81, 1.97)	7	0.49 (0.20, 1.19)
Monobromoacetic acid (MBAA)^*g*^	
> 0.04–10.63	8	1.17 (0.49, 2.77)	13	1.81 (0.85, 3.84)	1	0.66 (0.08, 5.13)
Dibromoacetic acid (DBAA)^*g*^
> 0.47–21.78	27	0.81 (0.50, 1.32)	28	1.00 (0.63, 1.61)	7	0.38 (0.14, 1.02)
DBP9
> 33.07–59.95	76	1.12 (0.61, 2.08)	58	1.33 (0.71, 2.48)	26	1.12 (0.42, 2.99)
> 59.95–79.13	87	1.46 (0.77, 2.77)	79	1.70 (0.88, 3.26)	26	1.02 (0.35, 2.96)
> 79.13–97.67	84	1.37 (0.71, 2.64)	71	1.64 (0.83, 3.26)	19	0.63 (0.21, 1.93)
> 97.67–181.59	58	0.67 (0.33, 1.35)	63	1.48 (0.73, 2.98)	24	0.79 (0.25, 2.49)
Note: CI, confidence interval; DBP9, sum of chloroform, BDCM, DBCM, bromoform, MCAA, DCAA, TCAA, MBAA, and DBAA; HAA5, sum of MCAA, DCAA, TCAA, MBAA, and DBAA; THM4, sum of chloroform, BDCM, DBCM, and bromoform; THMBr, sum of BDCM, DBCM, and bromoform. ^***a***^For each DBP metric, the referent for each model includes exposure scores of zero up to the lower bound of the lowest exposure category.^***b***^The numbers represent the case distribution across exposure groups before modeling.^***c***^Models adjusted for the type of water source and treatment, infant birth weight, health index (gestational diabetes, nongestational diabetes, chronic hypertension, gestational hypertension, and hydramnios/oligohydramnios), and other maternal reproductive risk factors.^***d***^Models adjusted for the type of water source and treatment, maternal marital status (married, including within 300 days before birth; not married), maternal education category, maternal race category, health index (gestational diabetes, non-gestational diabetes, chronic hypertension, gestational hypertension, and hydramnios/oligohydramnios), and other maternal reproductive risk factors.^***e***^Models adjusted for the type of water source and treatment, ZIP code–level income quartile, trimester prenatal care began (first, after first), category of prenatal care source payment, health index (gestational diabetes, nongestational diabetes, chronic hypertension, gestational hypertension, and hydramnios/oligohydramnios), and other maternal reproductive risk factors. ^***f***^Models also include adjustment for HAA5 concentrations.^***g***^Models also include adjustment for THM4 concentrations.						

Consistent elevated aORs were detected for VSDs and every DBP metric except chloroform, TCAA, and HAA5 (Table 4). aORs were comparable between VSDs and each THM4 (aOR range, 1.39–1.77) and DBP9 (aOR range, 1.33–1.70) quintile. Although not statistically significant, aORs smaller in magnitude were noted between VSDs and the highest BDCM tertile (aOR = 1.21; 95% CI: 0.79, 1.85), the upper THMBr quintile (aOR = 1.34; 95% CI: 0.73, 2.46), and the upper DCAA quartiles (aOR = 1.18; 95% CI: 0.65, 2.14). The strongest associations for VSDs and brominated DBPs were found for bromoform (aOR = 1.85; 95% CI: 1.20, 2.83), MBAA (aOR = 1.81; 95% CI: 0.85, 3.84), and DBCM (aOR = 1.54; 95% CI: 1.00, 2.37).

When we examined the most consistent associations (i.e., TOF and VSDs) for potential effect measure modification by infant sex, no discernible patterns were seen between HAA5 quintiles and TOF. Larger aORs were detected among females for TOF and DCAA, whereas males had higher aORs for TCAA exposures (see Table S2). The aORs for MBAA and VSDs were three times higher among males, with smaller increases detected among males for bromoform, DBCM, and MCAA (see Table S3). aORs for the remaining DBP measures (DBP9, THM4, THMBr, BDCM, and chloroform) were considerably larger among females, including some relationships that were largely null for VSDs in the main analysis (e.g., BDCM and chloroform).

## Discussion

Unlike a recent study and meta-analysis, we did not see any evidence for associations between first-trimester THM4 exposures and the All CVD group (Grazuleviciene et al. 2013; Nieuwenhuijsen et al. 2009). The strongest association that we detected for individual DBPs and All CVD was for dichotomized bromoform exposures > 0.26 μg/L (aOR = 1.43; 95% CI: 1.10, 1.86). This is higher than that of the only other study published to date to examine bromoform by Nieuwenhuijsen et al. (2008), who found an aOR of 1.18 (95% CI: 1.00, 1.39) for a subset of etiologically similar cardiac defects and bromoform levels ≥ 4 μg/L (vs. < 2 μg/L). Given that CVDs are a heterogeneous group of outcomes with different underlying mechanisms and etiologies, our primary focus was to examine individual CVDs and etiologically relevant groups in relation to DBPs.

We detected positive exposure–response relationships between TCAA exposure and pulmonary stenosis. The strongest associations that we detected were for the conotruncal defects including TGA and TOF, although the only statistically significant association for TGA was detected for bromoform exposures (aOR = 2.42; 95% CI: 1.12, 5.23). Stronger associations were detected between TOF and the upper exposure categories for TCAA, DCAA, and HAA5 (aOR range, 3.34–6.51) including exposure–response relationships for DCAA and HAA5. These findings are consistent with animal data showing CVDs following TCAA and DCAA exposures (Epstein et al. 1992; Johnson et al. 1998; Smith et al. 1989, 1992). The only epidemiological study of DBPs to examine conotruncal defects as a group saw some suggestion of increased risk only for THM4 exposures (aOR = 1.5; 95% CI: 0.67, 3.50 for 50–74 vs. 0 μg/L), although they did not have sufficient data to examine bromoform or HAA exposures (Shaw et al. 2003). A study in Norway examined TOF and THM4, but their data were hampered by very small cell sizes and a limited exposure contrast (Hwang et al. 2008). We also detected elevated aORs for ASDs and bromoform exposures (aOR = 1.56; 95% CI: 1.01, 2.43) and across every chloroform quintile (aOR range, 1.38–1.67). The magnitude of these associations are consistent with the only other study to examine ASDs, although that study was based on very small sample sizes across THM4 quartiles (Hwang et al. 2008).

Similar to a meta-analysis (OR = 1.59, 95% CI: 1.21, 2.07) by Nieuwenhuijsen et al. (2009), we found consistently elevated aORs (aOR range, 1.39–1.77) for VSD across all exposure THM4 quintiles (beginning at concentrations of 23.05 μg/L) with an aOR of 1.57 (95% CI: 0.70, 3.53) for high THM4 exposures (> 65.27 vs. ≤ 23.05 μg/L). In concordance with two previous VSD studies that evaluated THM4 exposures in the United Kingdom (aOR = 1.43; 95% CI: 1.0, 2.04 for > 60 vs. < 30 μg/L) and Norway (aOR = 1.81; 0.98–3.35 for > 20 vs. ≤ 4 μg/L), our studies add to the consistency reported in epidemiological studies of THMs and VSDs published to date (Hwang et al. 2008; Nieuwenhuijsen et al. 2008). We also saw consistent evidence of associations between VSD and various brominated DBP exposure metrics, with the strongest associations noted for bromoform, MBAA, and DBCM (aOR range; 1.54–1.85). The bromoform (aOR = 1.85; 95% CI: 1.20, 2.83) and VSD associations are stronger than those of the only study to examine bromoform exposures (aOR = 1.27; 95% CI: 0.89, 1.82) in relation to isolated VSDs (Nieuwenhuijsen et al. 2008). In our study, bromoform was consistently associated with elevated aORs for all the individual and group CVDs examined. These findings help inform the specificity of reported associations with DBPs and may explain some of the consistent results noted in previous studies for THM4 exposures.

A key study strength was our ability to evaluate numerous individual and summary DBP exposure metrics, because the limitation of examining THM4 and other surrogates is well established. This is the first study of CVDs to assess alternative DBP mixture surrogates including HAA5 and DBP9. The large sample size and sufficient DBP exposure gradients also enabled examination of individual birth defects in relation to low-exposure referents for various DBP metrics. Statistical power was limited for some less prevalent DBP metrics (e.g., MCAA and MBAA) and the rarest CVDs such as TGA and TOF; this may have precluded detection of statistically significant associations small in magnitude as well as exposure–response relationships. Although we acknowledge that some of the results for different CVD and DBP combinations that were examined may be due to chance, our study does help address specificity of causal associations that have been identified in the toxicological literature.

Based on the extensive number of available covariates, we adjusted for various confounders including strong maternal risk factors for CVDs as well as other exposures related to DBPs, such as water source and disinfection type. This is also the first birth defect study to develop multi-pollutant models to examine potential confounding by other DBP exposures. We used a matched case–control study design to increase statistical efficiency and to control for time-varying confounding. An additional strength of our population-based case–control study is the low risk of selection bias, because the cases and controls were both drawn from the same study base of all underlying births in Massachusetts. We also saw no evidence to suggest that the CVD cases that were not included due to missing data or other exclusion factors were disproportionately exposed to higher or lower DBP concentrations in drinking water.

One of the main limitations in many epidemiological studies of DBPs is the lack of individual-level exposure data which may better reflect internal dose. We relied on routinely monitored data that were collected at least quarterly for all water systems. Given the known seasonality detected for some DBPs such as the THMs, quarterly measures may not fully capture the extent of temporal variability that may influence exposure estimates. For example, the critical *in utero* exposure period for many of the CVDs is during the 3rd through 8th weeks of gestation, but we did not have samples that corresponded exactly with that time period. Thus, our use of first-trimester average DBP exposure may result in exposure misclassification, which we would expect to be nondifferential in nature. Residential mobility may also lead to exposure misclassification if the address reported at birth differed from the first-trimester residence. A review of 14 environmental epidemiological studies of pregnancy outcomes showed that most moves occurred during the second trimester among the 9–32% of pregnant women who reported moving residence (Bell and Belanger 2012). Previous research has shown that these moves are often short in distance, with only 8% of cases moving to a different county during the pregnancy (Bell and Belanger 2012; Khoury et al. 1988). This suggests that most moves during pregnancy often occur to residences that rely on the same water system. The impact of mobility on our study results is difficult to determine, but a previous study of DBPs and neural-tube defects reported stronger associations among mothers with confirmed residences at conception compared with the overall population of confirmed and unconfirmed residences (Klotz and Pyrch 1999).

In addition to uncaptured temporal variability, measurement error may result in exposure misclassification from the use of town-average DBP estimates to estimate individual exposures, because they do not include information on inter- and intraindividual variability in water use patterns. Town-average DBP concentrations from different sampling locations in water systems with considerable spatial variability may also not fully reflect residential values, although we are confident that our exposure assessment should largely capture relative categorical rankings of overall DBP exposures via drinking water. Nonetheless, we recognize that these potential sources of measurement error can lead to exposure misclassification, which may bias our results and distort any exposure–response relationships that may exist.

Massachusetts maintains an active statewide, population-based birth defect registry system to track birth defects in the 1st year following pregnancy. Therefore, we would expect minimal case underascertainment to have occurred for CVDs that occur up to 1 year, but are less certain about defects that are detected predominantly beyond the first year. There is some potential for outcome misclassification due to measurement error from the use of the ICD-9 codes (Cronk et al. 2003; Holmes and Westgate 2012; Strickland et al. 2008). Inaccuracies attendant with the use of ICD-9 codes can vary substantially by birth defect subtype and can lead to false positives and false negatives. ICD-9 codes have been shown to be good at classifying certain heart defects, such as TOF (100%), coarctation of the aorta (100%), and VSD (84%), whereas others such as ASDs (50%) and patent ductus arteriosus (22%) are less accurate (Frohnert et al. 2005). Other studies have also reported variability in false positive rates from 2% for TOF to 49% for TGA (Strickland et al. 2008). Some less severe cardiac defects, such as small holes in the heart (e.g., ASDs or VSDs), may spontaneously close or repair themselves during pregnancy or shortly after birth. Such defects may in fact go undetected, so our study population may be capturing fewer minor defects in general. We conducted a sensitivity analysis of the All CVD category excluding patent ductus arteriosus which, as noted above, is prone to misclassification. Following this restriction, comparable results were detected for bromoform and DCAA, with larger aORs found for the highest HAA5 exposure category (1.53 vs. 1.18) (see Table S4).

As with other epidemiological studies based on birth records, we cannot gauge the extent to which elective pregnancy terminations may be related to the prevalence of birth defects among aborted fetuses. Although elective terminations can result in underascertainment of cases detected at birth based on vital records data, CVDs are not often the medical reason why abortions are pursued. For example, the reported elective termination rate when detected prenatally is < 5% for ASDs, VSDs, TGA, and TOF (Boldt et al. 2002; Ethen and Canfield 2002; Papp et al. 1995; Stoll et al. 1993; Wren et al. 2000). Because our study population comprises only live births, we may not be capturing all birth defect cases in this population, including those that resulted in miscarriages. However, many CVDs, such as ASDs, often occur among live births (Botto et al. 2001; Forrester and Merz 2004; Garne et al. 2001).

CVDs are often idiopathic and likely involve multiple etiological factors including genetics, lifestyle factors, and other environmental determinants. We minimized the potential for false-positive associations between DBPs and CVDs through exclusion of chromosomal abnormalities which resulted in a more homogenous study population. We also minimized the influence of known cardiac birth defects risk factors such as rubella by use of statistical adjustment in the confounding analysis. CVDs also represent a wide range of types of malformations, some of which are simple or complex in nature. Complex CVDs, such as TOF, include a combination of CVDs diagnosed together. Because they may obscure some of the relationships that were examined for individual defects, we also conducted a sensitivity analysis to examine the impact of multiple birth defects. The sensitivity analysis was limited to CVDs with the strongest and most consistent associations. The aORs for isolated VSDs were slightly lower for the individual DBPs such as bromoform (1.42 vs. 1.85) and MBA (1.41 vs. 1.81), but were larger for the DBP mixture surrogates THM4 (2.74 vs. 1.57) and DBP9 (2.14 vs. 1.48) (see Table S5). In contrast to the main analysis, a positive exposure–response relationship for VSDs was detected for THM4 with consistent associations in the upper three quintiles (aOR range, 2.41–2.74). The associations for TOF were even stronger in magnitude for isolated TOF cases, where the aORs were from two to three times larger for the highest DCAA quartile (11.11 vs. 3.34), TCAA quartile (9.17 vs 3.89), and the HAA5 quintile (12.37 vs. 6.51) (see Table S6).

As noted earlier, we were able to evaluate numerous risk factors for birth defects from the comprehensive information available from the birth records. The reliance on birth record data, however, may limit the ability to fully consider some potential confounders such as vitamin use, body mass index (BMI), alcohol use, passive smoking, and other socioeconomic indicators. Alcohol consumption, for example, is an important risk factor for some birth defects, but the Massachusetts Department of Public Health had advised that the birth data on maternal alcohol consumption are considered of poor quality and of questionable validity. Thus, we did not include maternal alcohol use in the analyses. This is in contrast to reported smoking during pregnancy, which we have much more confidence in given that we previously demonstrated strong relationships between maternal cigarette use during pregnancy and different fetal growth measures (Rivera-Núñez and Wright 2013; Wright et al. 2004). Previous research also indicates good agreement with cotinine levels and self-reported maternal cigarette use during pregnancy (Searles Nielsen et al. 2014). Although we did not have reliable data for some potential CVD risk factors such as maternal alcohol use and prepregnancy BMI, we do not expect these to be strongly associated with DBP exposures in our study. If present at all, any bias would likely result in negative confounding if DBP exposures and alcohol consumption are inversely associated. Therefore, we would expect any residual confounding from this and other inversely associated covariates, such as obesity and BMI, to attenuate observed associations toward the null if they are not adjusted for or addressed in the study design phase. In addition, we did have data on maternal weight gain during pregnancy, which is likely related to obesity, BMI, and healthful behaviors during pregnancy. Thus, statistical adjustment for weight gain may indirectly control for some of the potential confounding from maternal BMI.

We adjusted for income in most of the logistic regression models, because we saw fairly consistent evidence of confounding by aggregate income levels based on census data for towns, census tracts, or ZIP codes. Because individual-level income data was not available, we recognize that residual confounding is possible if the aggregate measures resulted in misclassification. However, we saw little evidence of confounding by a socioeconomic index that combined aggregate and individual-level data, and our previous study found little evidence that aggregate socioeconomic indices were associated with DBP concentrations in Massachusetts public drinking water systems (Evans et al. 2013). Given this and our extensive confounding analysis including adjustment for other individual-level correlates of socioeconomic status (e.g., education, marital status, prenatal care source of payment), we would suspect that any potential residual confounding by income would have minimal impact on our results.

Although many of our results were null, we found consistent results for bromoform and every cardiac birth defect that was examined as well as increased risks for VSDs and different DBPs. Toxicological evidence lends credence to our study findings given that dose-dependent VSDs and conotruncal defects have been shown in rats following DCAA and TCAA exposures (Epstein et al. 1992; Johnson et al. 1998; Smith et al. 1989, 1992). Bromochloroacetonitrile has also been reported to cause CVDs in rats (Christ et al. 1995), but the haloacetonitriles have yet to be examined in an epidemiological study. This may be important given that bromochloroacetonitrile was shown to be highly correlated with some DBPs such as haloacetamides and the trihaloacetic acids (e.g., TCAA) in a study from the United Kingdom (Bond et al. 2015). Despite some findings that appear concordant with existing epidemiological and toxicological studies, more research is needed to further elucidate which DBPs or DBP mixtures may be responsible for the epidemiological associations reported to date.

## Conclusions

This is the first epidemiological study of birth defects and DBPs to examine several individual CVDs, different exposure surrogate mixtures (THM4, THMBr, HAA5, and DBP9), and various individual DBP species. Future analyses of CVDs and DBPs should expand upon this research and focus efforts to reduce exposure misclassification due to spatial and temporal variability including evaluation of smaller critical exposure windows and peak exposures. This may require more frequent distribution system sampling or temporal modeling/interpolation approaches using existing data. Residential-level sampling or more spatially representative exposure estimates (e.g., geographic information system–based approaches) would also help address spatial variability concerns.

There are fairly consistent results from epidemiological and toxicological studies for associations between DBP exposures and increased risk of some CVDs, especially VSDs. Because our study is only the second one to evaluate exposure to brominated DBPs, it adds some specificity to the potential risks that have been previously noted for THM4. However, further clarity on which of the co-occurring DBP species (or mixture combinations) to sample for and analyze is still needed and may benefit from additional toxicological studies and exposure assessment research. Given the ubiquitous nature of DBPs in treated drinking water, our findings have potential important public health ramifications. Thus, further delineation of the potential impact of *in utero* exposure to environmental teratogens would help inform intervention efforts to reduce exposures during critical windows of pregnancy.

## Supplemental Material

(188 KB) PDF

## References

[r1] Bell ML, Belanger K (2012). Review of research on residential mobility during pregnancy: consequences for assessment of prenatal environmental exposures.. J Expo Sci Environ Epidemiol.

[r2] Boldt T, Andersson S, Eronen M (2002). Outcome of structural heart disease diagnosed in utero.. Scand Cardiovasc J.

[r3] Bond T, Templeton MR, Mokhtar Kamal NH, Graham N, Kanda R (2015). Nitrogenous disinfection byproducts in English drinking water supply systems: occurrence, bromine substitution and correlation analysis.. Water Res.

[r4] Botto LD, Correa A, Erickson JD (2001). Racial and temporal variations in the prevalence of heart defects.. Pediatrics.

[r5] Bove FJ, Fulcomer MC, Klotz JB, Esmart J, Dufficy EM, Savrin JE (1995). Public drinking water contamination and birth outcomes.. Am J Epidemiol.

[r6] Brent RL, Beckman DA (1990). Environmental teratogens.. Bull N Y Acad Med.

[r7] Chisholm K Cook A Bower C Weinstein P 2008 Risk of birth defects in Australian communities with high levels of brominated disinfection by-products. Environ Health Perspect 116 1267 1273, doi:10.1289/ehp.10980 18795174 PMC2535633

[r8] Christ SA, Read EJ, Stober JA, Smith MK (1995). The developmental toxicity of bromochloroacetonitrile in pregnant Long–Evans rats.. Int J Environ Health Res.

[r9] Christian MS, York RG, Hoberman AM, Diener RM, Fisher LC (2001). Oral (drinking water) developmental toxicity studies of bromodichloromethane (BDCM) in rats and rabbits.. Int J Toxicol.

[r10] Cronk CE, Malloy ME, Pelech AN, Miller RE, Meyer SA, Cowell M (2003). Completeness of state administrative databases for surveillance of congenital heart disease.. Birth Defects Res A Clin Mol Teratol.

[r11] Epstein DL, Nolen GA, Randall JL, Christ SA, Read EJ, Stober JA (1992). Cardiopathic effects of dichloroacetate in the fetal Long-Evans rat.. Teratology.

[r12] Ethen MK, Canfield MA (2002). Impact of including elective pregnancy terminations before 20 weeks gestation on birth defect rates.. Teratology.

[r13] Evans AM, Wright JM, Meyer A, Rivera-Núñez Z (2013). Spatial variation of disinfection by-product concentrations: exposure assessment implications.. Water Res.

[r14] Forrester MB, Merz RD (2004). Descriptive epidemiology of selected congenital heart defects, Hawaii, 1986–1999.. Paediatr Perinat Epidemiol.

[r15] Frohnert BK, Lussky RC, Alms MA, Mendelsohn NJ, Symonik DM, Falken MC (2005). Validity of hospital discharge data for identifying infants with cardiac defects.. J Perinatol.

[r16] Garne E Stoll C Clementi M, Euroscan Group. 2001 Evaluation of prenatal diagnosis of congenital heart diseases by ultrasound: experience from 20 European registries. Ultrasound Obstet Gynecol 17 5 386 391 11380961 10.1046/j.1469-0705.2001.00385.x

[r17] Go AS, Mozaffarian D, Roger VL, Benjamin EJ, Berry JD, Borden WB (2013). Heart disease and stroke statistics—2013 update: a report from the American Heart Association.. Circulation.

[r18] Graves CG, Matanoski GM, Tardiff RG (2001). Weight of evidence for an association between adverse reproductive and developmental effects and exposure to disinfection by-products: a critical review.. Regul Toxicol Pharmacol.

[r19] Grazuleviciene R, Kapustinskiene V, Vencloviene J, Buinauskiene J, Nieuwenhuijsen MJ (2013). Risk of congenital anomalies in relation to the uptake of trihalomethane from drinking water during pregnancy.. Occup Environ Med.

[r20] Holmes LB, Westgate MN (2012). Using ICD-9 codes to establish prevalence of malformations in newborn infants.. Birth Defects Res A Clin Mol Teratol.

[r21] Hwang BF Jaakkola JJ Guo HR 2008 Water disinfection by-products and the risk of specific birth defects: a population-based cross-sectional study in Taiwan. Environ Health 7 23, doi:10.1186/1476-069X-7-23 18518952 PMC2467412

[r22] Johnson PD, Dawson BV, Goldberg SJ (1998). Cardiac teratogenicity of trichloroethylene metabolites.. J Am Coll Cardiol.

[r23] Khoury MJ, Stewart W, Weinstein A, Panny S, Lindsay P, Eisenberg M (1988). Residential mobility during pregnancy: implications for environmental teratogenesis.. J Clin Epidemiol.

[r24] Klotz JB, Pyrch LA (1999). Neural tube defects and drinking water disinfection by-products.. Epidemiology.

[r25] Kotelchuck M (1994). An evaluation of the Kessner Adequacy of Prenatal Care Index and a proposed Adequacy of Prenatal Care Utilization Index.. Am J Public Health.

[r26] Kurinczuk JJ, Hollowell J, Boyd PA, Oakley L, Brocklehurst P, Gray R (2010). The contribution of congenital anomalies to infant mortality. Inequalities in Infant Mortality Project Briefing Paper 4.. https://www.npeu.ox.ac.uk/downloads/files/infant-mortality/Infant-Mortality-Briefing-Paper-4.pdf.

[r27] Lee K, Khoshnood B, Chen L, Wall SN, Cromie WJ, Mittendorf RL (2001). Infant mortality from congenital malformations in the United States, 1970–1997.. Obstet Gynecol.

[r28] Nieuwenhuijsen MJ Martinez D Grellier J Bennett J Best N Iszatt N 2009 Chlorination disinfection by-products in drinking water and congenital anomalies: review and meta-analyses. Environ Health Perspect 117 1486 1493, doi:10.1289/ehp.0900677 20019896 PMC2790500

[r29] Nieuwenhuijsen MJ Toledano MB Bennett J Best N Hambly P de Hoogh C 2008 Chlorination disinfection by-products and risk of congenital anomalies in England and Wales. Environ Health Perspect 116 216 222, doi:10.1289/ehp.10636 18288321 PMC2235225

[r30] Papp Z, Tóth-Pál E, Papp C, Tóth Z, Szabó M, Veress L (1995). Impact of prenatal mid-trimester screening on the prevalence of fetal structural anomalies: a prospective epidemiological study.. Ultrasound Obstet Gynecol.

[r31] Randall JL, Christ SA, Horton-Perez P, Nolen GA, Read EJ, Smith MK (1991). Developmental effects of 2-bromoacetic acid in the Long-Evans rat [Abstract P122].. Teratology.

[r32] Righi E, Bechtold P, Tortorici D, Lauriola P, Calzolari E, Astolfi G (2012). Trihalomethanes, chlorite, chlorate in drinking water and risk of congenital anomalies: a population-based case-control study in Northern Italy.. Environ Res.

[r33] Rivera-Núñez Z, Wright JM (2013). Association of brominated trihalomethane and haloacetic acid exposure with fetal growth and preterm delivery in Massachusetts.. J Occup Environ Med.

[r34] Ruddick JA, Villeneuve DC, Chu I, Valli VE (1983). A teratological assessment of four trihalomethanes in the rat.. J Environ Sci Health B.

[r35] Searles Nielsen S, Dills RL, Glass M, Mueller BA (2014). Accuracy of prenatal smoking data from Washington State birth certificates in a population-based sample with cotinine measurements.. Ann Epidemiol.

[r36] Shaw GM, Ranatunga D, Quach T, Neri E, Correa A, Neutra RR (2003). Trihalomethane exposures from municipal water supplies and selected congenital malformations.. Epidemiology.

[r37] Smith MK, Randall JL, Read EJ, Stober JA (1989). Teratogenic activity of trichloroacetic acid in the rat.. Teratology.

[r38] Smith MK, Randall JL, Read EJ, Stober JA (1992). Developmental toxicity of dichloroacetate in the rat.. Teratology.

[r39] Stoll C, Alembik Y, Dott B, Roth PM, De Geeter B (1993). Evaluation of prenatal diagnosis of congenital heart disease.. Prenat Diagn.

[r40] Strickland MJ, Riehle-Colarusso TJ, Jacobs JP, Reller MD, Mahle WT, Botto LD (2008). The importance of nomenclature for congenital cardiac disease: implications for research and evaluation.. Cardiol Young.

[r41] Wren C, Richmond S, Donaldson L (2000). Temporal variability in birth prevalence of cardiovascular malformations.. Heart.

[r42] Wright JM Schwartz J Dockery DW 2004 The effect of disinfection by-products and mutagenic activity on birth weight and gestational duration. Environ Health Perspect 112 920 925, doi:10.1289/ehp.6779 15175183 PMC1242023

