# Supplementary material for: Disinfection By-Product Exposures and the Risk of Specific Cardiac Birth Defects
Source: Environ Health Perspect. 2016 Aug 12;125(2):269–77. doi: 10.1289/EHP103 (PMC5289901; doi:10.1289/EHP103)
Supplement: (188 KB) PDF [file EHP103.s001.acco.pdf]

Environ Health Perspect
DOI: 10.1289/EHP103

Note to readers with disabilities: *EHP* strives to ensure that all journal content is accessible to all readers. However, some figures and Supplemental Material published in *EHP* articles may not conform to 508 standards due to the complexity of the information being presented. If you need assistance accessing journal content, please contact ehp508@niehs.nih.gov. Our staff will work with you to assess and meet your accessibility needs within 3 working days.

Supplemental Material

Disinfection By-Product Exposures and the Risk of Specific Cardiac Birth Defects

J. Michael Wright, Amanda Evans, John A. Kaufman, Zorimar Rivera-Núñez, and Michael G. Narotsky

Table of Contents

Table S1. Spearman Correlation Coefficients for Disinfection By-Product Metrics

Table S2. Adjusted Odds Ratios (aORs) between Disinfection By-Product (DBP) Exposures and Tetralogy of Fallot Stratified by Sex

Table S3. Adjusted Odds Ratios (aORs) between Disinfection By-Product (DBP) Exposures and Ventricular Septal Defects Stratified by Sex

Table S4. Adjusted Odds Ratios (aORs) between Disinfection By-Product (DBP) Exposures and All Cardiovascular Defects Excluding Patent Ductus Arteriosus

Table S5. Adjusted Odds Ratios (aORs) between Disinfection By-Product (DBP) Exposures and Isolated Ventricular Septal Defect Cases

Table S6. Adjusted Odds Ratios (aORs) between Disinfection By-Product (DBP) Exposures and Isolated Tetralogy of Fallot Cases

Table S1. Spearman Correlation Coefficients for Disinfection By-Product Metrics

	DBP9	HAA5	THM4	THMBr	BDCM	DBCM	TCM	TCAA	DCAA	TBM	MBAA	MCAA	DBAA
**DBP9**	1.00												
**HAA5**	0.90	1.00											
**THM4**	0.95	0.74	1.00										
**THMBr**	0.40	0.25	0.50	1.00									
**BDCM**	0.54	0.37	0.63	0.96	1.00								
**DBCM**	0.05	-0.05	0.13	0.85	0.72	1.00							
**TCM**	0.94	0.76	0.97	0.33	0.47	-0.02	1.00						
**TCAA**	0.89	0.96	0.77	0.24	0.37	-0.08	0.79	1.00					
**DCAA**	0.84	0.93	0.70	0.23	0.34	-0.06	0.74	0.85	1.00				
**TBM**	-0.25	-0.28	-0.20	0.39	0.20	0.57	-0.30	-0.32	-0.28	1.00			
**MBAA**	0.03	0.04	0.02	0.03	0.03	0.04	0.02	0.04	0.02	0.03	1.00		
**MCAA**	0.50	0.57	0.42	0.26	0.30	0.07	0.42	0.46	0.55	-0.13	0.04	1.00	
**DBAA**	-0.12	-0.09	-0.11	0.31	0.19	0.41	-0.18	-0.17	-0.07	0.54	0.15	0.01	1.00

Note: DBP9 = sum of chloroform (TCM), bromodichloromethane (BDCM), dibromochloromethane (DBCM), bromoform (TBM), monochloroacetic acid (MCAA), dichloroacetic acid (DCAA), trichloroacetic acid (TCAA), bromoacetic acid (MBAA), and dibromoacetic acid (DBAA); HAA5 = sum of MCAA, DCAA, TCAA, MBAA, and DBAA; THM4 = sum of TCM, BDCM, DBCM, and TBM; THMBr = sum of BDCM, DBCM, and TBM

Table S2. Adjusted Odds Ratios (aORs) between Disinfection By-Product (DBP) Exposures and Tetralogy of Fallot Stratified by Sex

DBP Metrics (μg/L)	Cases (n)[a]	Total Population aOR (95% CI)[b]	Male aOR (95% CI)[b]	Female aOR (95% CI)[b]
THM4[c]				
≤23.05	15	REF	REF	REF
>23.05–38.05	17	0.32 (0.10, 1.05)	1.52 (0.17, 13.13)	0.15 (0.01, 1.64)
>38.05–50.41	19	0.51 (0.15, 1.74)	3.02 (0.30, 30.43)	0.16 (0.02, 1.47)
>50.41–65.27	19	0.31 (0.08, 1.21)	1.53 (0.14, 16.62)	0.22 (0.02, 2.12)
>65.27–125.32	16	0.19 (0.04, 0.88)	1.06 (0.09, 12.09)	0.05 (0.00, 0.72)
THMBr[c]				
≤4.17	15	REF	REF	REF
>4.17–6.04	25	1.00 (0.34, 2.98)	0.29 (0.04, 2.10)	5.21 (0.58, 46.62)
>6.04–7.80	12	0.23 (0.06, 0.91)	0.19 (0.02, 1.81)	0.45 (0.03, 7.88)
>7.80–11.51	18	0.45 (0.14, 1.39)	0.17 (0.02, 1.32)	1.64 (0.22, 12.36)
>11.51–42.48	16	0.26 (0.09, 0.76)	0.27 (0.04, 1.83)	0.28 (0.03, 2.70)
Chloroform[c]				
≤12.07	15	REF	REF	REF
>12.07–29.99	14	0.50 (0.16, 1.53)	1.06 (0.12, 9.48)	0.19 (0.02, 2.24)
>29.99–42.17	21	0.76 (0.22, 2.69)	1.67 (0.17, 15.96)	0.23 (0.02, 3.32)
>42.17–55.41	22	0.81 (0.20, 3.29)	1.91 (0.14, 26.80)	0.29 (0.02, 3.98)
>55.41–98.99	14	0.33 (0.07, 1.64)	0.50 (0.03, 7.73)	0.06 (0.00, 1.46)
Bromodichloromethane (BDCM)[c]				
≤4.95	34	REF	REF	REF
>4.95–7.55	24	0.49 (0.19, 1.24)	0.73 (0.17, 3.09)	0.53 (0.10, 2.72)
>7.55–37.38	28	0.38 (0.17, 0.82)	0.76 (0.24, 2.44)	0.30 (0.06, 1.40)
Dibromochloromethane (DBCM)[c]				
≤3.93	79	REF	REF	REF
>3.93–14.53	7	0.54 (0.20, 1.41)	0.34 (0.07, 1.55)	0.36 (0.05, 2.79)
Bromoform[c]				
≤0.26	76	REF	REF	REF
>0.26–7.06	10	1.14 (0.45, 2.87)	0.59 (0.15, 2.33)	3.00 (0.34, 26.50)

Table S2. Adjusted Odds Ratios (aORs) between Disinfection By-Product (DBP) Exposures and Tetralogy of Fallot Stratified by Sex (continued)

DBP Metrics (µg/L)	Cases (*n*)[a]	Total Population aOR (95% CI)[b]	Male aOR (95% CI)[b]	Female aOR (95% CI)[b]
HAA5[d]				
≤8.17	11	REF	REF	REF
>8.17–19.33	21	2.13 (0.53, 8.65)	2.59 (0.13, 50.10)	1.36 (0.11, 16.16)
>19.33–25.79	21	4.98 (1.02, 24.35)	8.27 (0.25, 276.35)	6.14 (0.32, 118.72)
>25.79–33.97	17	5.88 (1.06, 32.57)	2.95 (0.08, 114.41)	7.57 (0.30, 192.35)
>33.97–100.00	16	6.51 (1.23, 34.59)	4.79 (0.16, 142.97)	2.97 (0.14, 62.87)
Trichloroacetic acid (TCAA)[d]				
≤5.23	15	REF	REF	REF
>5.23–11.09	28	2.72 (0.91, 8.13)	5.79 (0.60, 55.49)	3.35 (0.35, 32.18)
>11.09–16.38	22	4.30 (1.09, 16.88)	6.82 (0.55, 83.94)	2.97 (0.20, 44.62)
>16.38–73.39	19	3.89 (0.97, 15.66)	5.92 (0.51, 68.40)	2.25 (0.13, 39.31)
Dichloroacetic acid (DCAA)[d]				
≤5.18	17	REF	REF	REF
>5.18–10.44	22	1.39 (0.50, 3.88)	4.09 (0.52, 31.94)	0.87 (0.07, 11.29)
>10.44–13.85	24	3.08 (0.92, 10.34)	2.31 (0.27, 19.90)	7.45 (0.34, 164.24)
>13.85–38.89	21	3.34 (0.90, 12.43)	1.54 (0.11, 20.85)	26.35 (0.94, 737.88)
Monochloroacetic acid (MCAA)[d]				
≤1.53	80	REF	REF	REF
>1.53–62.39	4	0.83 (0.27, 2.54)	0.59 (0.09, 3.78)	0.47 (0.05, 4.67)
Monobromoacetic acid (MBAA)[d]				
≤0.04	81	REF	REF	REF
>0.04–10.63	3	0.80 (0.08, 7.48)	0.53 (0.01, 20.59)	N/E
Dibromoacetic acid (DBAA)[d]				
≤0.47	77	REF	REF	REF
>0.47–21.78	7	0.59 (0.23, 1.54)	0.64 (0.12, 3.38)	0.24 (0.03, 2.22)

Table S2. Adjusted Odds Ratios (aORs) between Disinfection By-Product (DBP) Exposures and Tetralogy of Fallot Stratified by Sex (continued)

DBP Metrics (μg/L)	Cases (*n*)[a]	Total Population aOR (95% CI)[b]	Male aOR (95% CI)[b]	Female aOR (95% CI)[b]
DBP9				
≤33.07	13	REF	REF	REF
>33.07–59.95	17	0.80 (0.24, 2.63)	1.70 (0.19, 14.97)	0.97 (0.09, 10.84)
>59.95–79.13	24	1.15 (0.32, 4.08)	2.62 (0.31, 22.08)	2.95 (0.20, 42.62)
>79.13–97.67	18	1.39 (0.39, 5.01)	4.25 (0.43, 42.28)	0.26 (0.02, 4.49)
>97.67–181.59	14	0.94 (0.23, 3.79)	0.74 (0.07, 7.34)	0.55 (0.03, 9.92)

Note: *n* = sample size; aOR = adjusted odds ratios; CI = confidence interval; REF = referent; THM4 = sum of chloroform, BDCM, DBCM, and bromoform; THMBr = sum of BDCM, DBCM, and bromoform; N/E = not estimable; HAA5 = sum of MCAA, DCAA, TCAA, MBAA, and DBAA; DBP9 = sum of chloroform, BDCM, DBCM, bromoform, MCAA, DCAA, TCAA, MBAA, and DBAA.

[a]The numbers represent the TOF case distribution across exposure groups prior to modeling and stratification.

[b]Models adjusted for the type of water source and treatment, infant birth weight, ZIP code-level income quartile, trimester prenatal care began (first, after first), health index (gestational diabetes, non-gestational diabetes, chronic hypertension, gestational hypertension, and hydramnios/oligohydramnios), and other maternal reproductive risk factors.

[c]Models also include adjustment for HAA5 concentrations.

[d]Models also include adjustment for THM4 concentrations.

Table S3. Adjusted Odds Ratios (aORs) between Disinfection By-Product (DBP) Exposures and Ventricular Septal Defects Stratified by Sex

DBP Metrics (μg/L)	Cases (*n*)[a]	Total Population aOR (95% CI)[b]	Male aOR (95% CI)[b]	Female aOR (95% CI)[b]
THM4[c]				
≤23.05	62	REF	REF	REF
>23.05–38.05	61	1.39 (0.72, 2.69)	1.85 (0.49, 6.93)	1.15 (0.49, 2.71)
>38.05–50.41	80	1.77 (0.87, 3.59)	2.47 (0.60, 10.11)	1.69 (0.66, 4.34)
>50.41–65.27	73	1.70 (0.80, 3.63)	1.93 (0.43, 8.62)	1.60 (0.59, 4.29)
>65.27–125.32	58	1.57 (0.70, 3.53)	1.35 (0.29, 6.37)	2.08 (0.71, 6.12)
THMBr[c]				
≤4.17	67	REF	REF	REF
>4.17–6.04	56	0.81 (0.39, 1.68)	0.39 (0.12, 1.26)	1.70 (0.57, 5.04)
>6.04–7.80	73	0.79 (0.41, 1.55)	0.48 (0.17, 1.38)	1.72 (0.63, 4.69)
>7.80–11.51	55	0.89 (0.47, 1.69)	0.54 (0.20, 1.45)	1.96 (0.73, 5.23)
>11.51–42.48	82	1.34 (0.73, 2.46)	0.96 (0.37, 2.46)	2.52 (0.99, 6.42)
Chloroform[c]				
≤12.07	66	REF	REF	REF
>12.07–29.99	61	1.05 (0.58, 1.90)	1.18 (0.38, 3.69)	1.06 (0.48, 2.34)
>29.99–42.17	74	0.86 (0.43, 1.72)	0.79 (0.22, 2.88)	0.90 (0.34, 2.35)
>42.17–55.41	75	1.00 (0.47, 2.12)	0.75 (0.18, 3.09)	1.04 (0.38, 2.84)
>55.41–98.99	57	0.79 (0.35, 1.80)	0.32 (0.07, 1.43)	1.43 (0.48, 4.22)
Bromodichloromethane (BDCM)[c]				
≤4.95	105	REF	REF	REF
>4.95–7.55	112	0.81 (0.50, 1.31)	0.76 (0.34, 1.71)	1.01 (0.51, 2.01)
>7.55–37.38	116	1.21 (0.79, 1.85)	1.07 (0.53, 2.17)	1.51 (0.84, 2.70)
Dibromochloromethane (DBCM)[c]				
≤3.93	293	REF	REF	REF
>3.93–14.53	40	1.54 (1.00, 2.37)	2.17 (1.06, 4.46)	1.61 (0.87, 2.96)
Bromoform[c]				
≤0.26	290	REF	REF	REF
>0.26–7.06	43	1.85 (1.20, 2.83)	2.23 (1.10, 4.52)	1.88 (1.01, 3.52)

Table S3. Adjusted Odds Ratios (aORs) between Disinfection By-Product (DBP) Exposures and Ventricular Septal Defects Stratified by Sex (continued)

DBP Metrics (µg/L)	Cases (*n*)[a]	Total Population aOR (95% CI)[b]	Male aOR (95% CI)[b]	Female aOR (95% CI)[b]
HAA5[d]				
≤8.17	69	REF	REF	REF
>8.17–19.33	50	0.66 (0.37, 1.20)	0.75 (0.26, 2.16)	0.49 (0.21, 1.12)
>19.33–25.79	74	1.00 (0.51, 1.94)	0.74 (0.22, 2.52)	0.85 (0.34, 2.13)
>25.79–33.97	63	1.01 (0.49, 2.08)	1.65 (0.47, 5.79)	0.39 (0.13, 1.12)
>33.97–100.00	78	1.02 (0.49, 2.12)	1.23 (0.33, 4.64)	0.62 (0.22, 1.75)
Trichloroacetic acid (TCAA)[d]				
≤5.23	80	REF	REF	REF
>5.23–11.09	81	0.90 (0.55, 1.50)	1.18 (0.47, 2.91)	0.63 (0.31, 1.27)
>11.09–16.38	83	0.81 (0.44, 1.51)	1.01 (0.34, 3.00)	0.46 (0.19, 1.10)
>16.38–73.39	86	0.79 (0.41, 1.50)	1.08 (0.34, 3.46)	0.43 (0.18, 1.08)
Dichloroacetic acid (DCAA)[d]				
≤5.18	78	REF	REF	REF
>5.18–10.44	72	0.84 (0.51, 1.39)	0.51 (0.21, 1.19)	0.79 (0.39, 1.59)
>10.44–13.85	92	1.28 (0.72, 2.25)	1.02 (0.41, 2.53)	1.19 (0.52, 2.73)
>13.85–38.89	88	1.18 (0.65, 2.14)	1.14 (0.45, 2.91)	0.83 (0.35, 1.95)
Monochloroacetic acid (MCAA)[d]				
≤1.53	295	REF	REF	REF
>1.53–62.39	35	1.27 (0.81, 1.97)	1.47 (0.72, 2.99)	1.06 (0.55, 2.04)
Monobromoacetic acid (MBAA)[d]				
≤0.04	317	REF	REF	REF
>0.04–10.63	13	1.81 (0.85, 3.84)	6.56 (1.60, 26.83)	1.92 (0.60, 6.16)
Dibromoacetic acid (DBAA)[d]				
≤0.47	302	REF	REF	REF
>0.47–21.78	28	1.00 (0.63, 1.61)	1.09 (0.52, 2.28)	1.08 (0.56, 2.10)

Table S3. Adjusted Odds Ratios (aORs) between Disinfection By-Product (DBP) Exposures and Ventricular Septal Defects Stratified by Sex (continued)

DBP Metrics (μg/L)	Cases (n)[a]	Total Population aOR (95% CI)[b]	Male aOR (95% CI)[b]	Female aOR (95% CI)[b]
DBP9				
≤33.07	63	REF	REF	REF
>33.07–59.95	58	1.33 (0.71, 2.48)	1.13 (0.36, 3.48)	1.29 (0.57, 2.91)
>59.95–79.13	79	1.70 (0.88, 3.26)	2.23 (0.68, 7.32)	1.64 (0.69, 3.91)
>79.13–97.67	71	1.64 (0.83, 3.26)	1.45 (0.43, 4.91)	1.37 (0.55, 3.40)
>97.67–181.59	63	1.48 (0.73, 2.98)	1.07 (0.32, 3.62)	1.49 (0.56, 3.94)

Note: n = sample size; aOR = adjusted odds ratio; CI = confidence interval; REF = referent; THM4 = sum of chloroform, BDCM, DBCM, and bromoform; THMBr = sum of BDCM, DBCM, and bromoform; HAA5 = sum of MCAA, DCAA, TCAA, MBAA, and DBAA; DBP9 = sum of chloroform, BDCM, DBCM, bromoform, MCAA, DCAA, TCAA, MBAA, and DBAA.

[a]The numbers represent the case distribution across exposure groups prior to modeling and stratification.

[b]Models adjusted for the type of water source and treatment, maternal marital status (married, including within 300 days prior to birth; not married), maternal education category, maternal race category, health index (gestational diabetes, non-gestational diabetes, chronic hypertension, gestational hypertension, and hydramnios/oligohydramnios), and other maternal reproductive risk factors.

[c]Models also include adjustment for HAA5 concentrations.

[d]Models also include adjustment for THM4 concentrations.

Table S4. Adjusted Odds Ratios (aORs) between Disinfection By-Product (DBP) Exposures and All Cardiovascular Defects Excluding Patent Ductus Arteriosus

**DBP Metrics (µg/L)**	**Cases (*n*)[a]**	**aOR (95% CI)[b]**
Bromoform[c]		
≤0.26	532	REF
>0.26–7.06	79	1.50 (1.10, 2.04)
HAA5[d]		
≤8.17	118	REF
>8.17–19.33	111	1.09 (0.71, 1.68)
>19.33–25.79	153	1.57 (0.97, 2.55)
>25.79–33.97	111	1.24 (0.72, 2.12)
>33.97–100.00	119	1.53 (0.90, 2.61)
Dichloroacetic acid (DCAA)[d]		
≤5.18	142	REF
>5.18–10.44	150	1.08 (0.76, 1.53)
>10.44–13.85	169	1.26 (0.83, 1.89)
>13.85–38.89	141	1.29 (0.84, 1.99)

Note: *n* = sample size; aOR = adjusted odds ratio; CI = confidence interval; REF = referent; HAA5 = sum of monochloroacetic acid, DCAA, trichloroacetic acid, monobromoacetic acid, and dibromoacetic acid.

[a]The numbers represent the case distribution across exposure groups prior to modeling.

[b]Models adjusted for the type of water source and treatment, health index (gestational diabetes, non-gestational diabetes, chronic hypertension, gestational hypertension, and hydramnios/oligohydramnios), and other maternal reproductive risk factors.

[c]Model also includes adjustment for HAA5 concentrations.

[d]Models also include adjustment for THM4 (sum of chloroform, bromodichloromethane, dibromochloromethane, and bromoform) concentrations.

Table S5. Adjusted Odds Ratios (aORs) between Disinfection By-Product (DBP) Exposures and Isolated Ventricular Septal Defect Cases

DBP Metrics (μg/L)	Cases (n)[a]	aOR (95% CI)[b]
THM4[c]		
≤23.05	22	REF
>23.05–38.05	12	1.17 (0.39, 3.57)
>38.05–50.41	21	2.41 (0.74, 7.82)
>50.41–65.27	23	2.46 (0.66, 9.21)
>65.27–125.32	23	2.74 (0.67, 11.22)
Dibromochloromethane (DBCM)[c]		
≤3.93	89	REF
>3.93–14.53	12	1.07 (0.48, 2.36)
Bromoform[c]		
≤0.26	87	REF
>0.26–7.06	14	1.42 (0.68, 2.98)
Monobromoacetic acid (MBAA)[d]		
≤0.04	97	REF
>0.04–10.63	3	1.41 (0.16, 12.43)
DBP9		
≤33.07	22	REF
>33.07–59.95	14	1.32 (0.46, 3.75)
>59.95–79.13	21	2.08 (0.68, 6.42)
>79.13–97.67	19	1.37 (0.41, 4.63)
>97.67–181.59	25	2.14 (0.66, 6.98)

Note: n = sample size; aOR = adjusted odds ratio; CI = confidence interval; REF = referent; THM4 = sum of chloroform, BDCM, DBCM, and bromoform; DBP9 = sum of chloroform, bromodichloromethane (BDCM), DBCM, bromoform, monochloroacetic acid, dichloroacetic acid, trichloroacetic acid, MBAA, and dibromoacetic acid.

[a]The numbers represent the case distribution across exposure groups prior to modeling.

[b]Models adjusted for water source and treatment type, maternal education category, maternal marital status (married, including within 300 days prior to birth; not married), maternal race category, health index (gestational diabetes, non-gestational diabetes, chronic hypertension, gestational hypertension, and hydramnios/oligohydramnios), and other maternal reproductive risk factors.

[c]Models also include adjustment for HAA5 (sum of MCAA, DCAA, TCAA, MBAA, and DBAA) concentrations.

[d]Model also includes adjustment for THM4 concentrations.

Table S6. Adjusted Odds Ratios (aORs) between Disinfection By-Product (DBP) Exposures and Isolated Tetralogy of Fallot Cases

**DBP Metrics (μg/L)**	**Cases (*n*)[a]**	**aOR (95% CI)[b]**
HAA5		
≤8.17	4	REF
>8.17–19.33	6	2.64 (0.26, 26.86)
>19.33–25.79	9	4.90 (0.40, 60.50)
>25.79–33.97	6	3.61 (0.21, 63.15)
>33.97–100.00	7	12.37 (0.75, 204.11)
Dichloroacetic acid (DCAA)		
≤5.18	6	REF
>5.18–10.44	6	1.44 (0.27, 7.54)
>10.44–13.85	7	2.77 (0.34, 22.70)
>13.85–38.89	11	11.11 (1.17, 105.86)
Trichloroacetic acid (TCAA)		
≤5.23	5	REF
>5.23– 11.09	8	6.30 (0.85, 46.84)
>11.09–16.38	10	12.07 (1.02, 143.10)
>16.38–73.39	7	9.17 (0.65, 129.15)

Note: *n* = sample size; aOR = adjusted odds ratio; CI = confidence interval; REF = referent; HAA5 = sum of monochloroacetic acid, DCAA, TCAA, monobromoacetic acid, and dibromoacetic acid.

[a]The numbers represent the case distribution across exposure groups prior to modeling.

[b]Models adjusted for water source and treatment type, trimester prenatal care began (first, after first), birth weight, zip code level income, other maternal reproductive risk factors, health index (gestational diabetes, non-gestational diabetes, chronic hypertension, gestational hypertension, and hydramnios/oligohydramnios), and THM4 (sum of chloroform, bromodichloromethane, dibromochloromethane, and bromoform) concentrations.
